# *Momordica cymbalaria* fruit extract attenuates high-fat diet-induced obesity and diabetes in C57BL/6 mice

**DOI:** 10.22038/IJBMS.2018.29354.7095

**Published:** 2018-10

**Authors:** Puttanarasaiah Mahesh Kumar, Marikunte V Venkataranganna, Kirangadur Manjunath, Gollapalle Lakshminarayanashastry Viswanatha, Godavarthi Ashok

**Affiliations:** 1 Department of Biotechnology, Padmashree Institute of Information Sciences, Bengaluru, Karnataka, India; 2 Connexios Life Sciences Pvt. Ltd., Bengaluru, Karnataka, India; 3 Department of Microbiology and Biotechnology, Bangalore University, Bengaluru, Karnataka, India; 4 Independent Researcher, Kengeri, Bangalore, Karnataka, India; 5 Radiant Research Services Pvt. Ltd., Bengaluru, Karnataka, India

**Keywords:** C57BL/6 mice, Diabetes, Herbal medicine, High-fat diet, Insulin resistance, *Momordica cymbalaria*

## Abstract

**Objective(s):**

The present study was aimed to evaluate the effect of methanolic fruit extract of *Momordica cymbalaria* (MeMC) against high-fat diet-induced obesity and diabetes in C57BL/6 mice.

**Materials and Methods:**

In the present study, six weeks old male C57BL/6 mice were divided into four groups. G-1 and G-2 served as lean control and HFD control, G-3 and G-4 received MeMC 25 and 50 mg/kg, BW doses; all the treatments were given for a period of 11 weeks. The parameters such as body weight, fasting blood glucose, insulin, cholesterol, free fatty acid, and oral glucose tolerance tests were performed, further, at the end of the study fasting body weight, and weights of organs such as the liver, heart, and adipose tissue were measured and the liver tissue was subjected to histopathology evaluation, and insulin resistance was expressed as HOMA-IR index.

**Results:**

The high-fat diet fed C57 mice showed significant elevation of body weight (*P*<0.01), blood glucose (*P*<0.01), insulin (*P*<0.01), cholesterol (*P*<0.01), free fatty acid (*P*<0.01), and HOMA-IR index (*P*<0.01) along with significant elevation of all organ weights and reduction in oral glucose tolerance (*P*<0.01) and brown adipose weight (*P*<0.01). The histopathology showed significant fatty infiltration and hypertrophy of hepatocytes. Interestingly, MeMC (50 mg/kg) alleviated all the HFD-induced perturbances significantly. Further, the HPLC analysis of MeMC revealed the presence of gallic acid and rutin as chief ingredients.

**Conclusion:**

MeMC possesses potent antidiabetic activity and ameliorates insulin resistance in HFD diet fed C57 mice.

## Introduction

Diabetes mellitus is a metabolic disorder associated with an abnormal increase in blood glucose levels, perhaps due to defects in insulin secretions, and/or insulin action, and/or both, which is accompanied by one or more symptoms such as hyperglycemia, polyuria, polydipsia, and polyphagia (1). Worldwide the incidence of diabetes is increasing at an alarming rate, and it is estimated that India, China, and America will have the largest population suffering from diabetes by 2030 (2). In recent times, diabetes and associated complications such as neuropathy, nephropathy, cardiomyopathy, retinopathy, and micro and macrovascular complications have got immense attention, due to lack of safe and effective medication for the therapeutic management of these complications (3). Presently many synthetic classes of drugs are available in the market for the management of diabetes, however, the potential side/adverse effects associated with these medications upon long-term use are the major limitations (3). In this prospect, the plant-based medicines are thought to have a better edge over synthetic drugs in terms of efficacy and safety, hence many scientists are aiming at herbal therapeutic agents for the management of diabetes and related complications (4-6). In support of this, in literature many plants have been scientifically studied and reported to possess potential antidiabetic activity, such as *Acacia arabica* (7), *Aegle marmelos* (8), *Allium cepa* (9), *Azadirachta indica* (10-11)**, ***Boerhavia diffusa* (12), *Musa sapientum* (13-14), *Terminalia belerica* (15), *Withania somnifera* (16), and so on. In this context, *Momordica cymbalaria* belonging to family Cucurbitaceae, has been scientifically well demonstrated to possess various biological activities such as antidiabetic (17-19), antioxidant (20), antiulcer (21), antihyperlipidemic (22), anticonvulsant (23), neuroprotective (24), etc. In our previous study, we have evaluated the antidiabetic activity of methanolic fruit extract of *Momordica cymbalaria* (MeMC)* in* *vitro* using L6 myotubes; we found that MeMC is very potent in enhancing glucose uptake in L6 myotubes, through enhanced expression of adiponectin, leptin, PPAR-γ, and GLUT-4 genes (25-26). Further, consistent with our studies, various parts of *M. cymbalaria* have been reported to possess potential hypoglycemic and anti-diabetic activity (17-19). However, to date, MeMC has not been evaluated against diet-induced diabetes. With this background, we thought to evaluate the effect of MeMC on HFD-induced diabetes and insulin resistance in C57BL/6 mice.

## Materials and Methods

***Drugs and chemicals***

The solvent used for HPLC analysis was of HPLC grade and procured from HiMedia Laboratories Pvt Ltd (Mumbai, India), Biochemical kits were procured from ERBA diagnostic (Mannheim GMBH, Germany) and all solvents used for extraction were of analytical grade and purchased from local firms.

***Collection, processing, and extraction of Plant material***

The fruits of *M. cymbalaria* were purchased from the Hospete market (Bellary District, Karnataka, India), during the months of March to May 2013, considering the seasonal conditions for obtaining the maximum yield of phytoconstituents. The plant material was identified and authenticated by Dr K Madhava Chetty, Professor of Botany, Sri Venkateshwara University, Tirupati. The plant material was processed and extracted as per the previously mentioned procedure. 

***HPLC analysis of methanolic fruit extract of Momordica cymbalaria***

The Shimadzu LC-10 ATVP system equipped with a UV detector (280 nm wavelength) and Chromtech N 2000 software were used for the current study (Shimadzu Analytical (India) Pvt. Ltd., New Delhi, India). The chromatographic conditions were as follows, gradient elution at a flow rate of 1.5 ml/min was employed on a symmetry reverse phase C18 column (250 mm x4. 6 mm, ID, 5 µm particle size) at ambient temperature. 

The mobile phase consisted of methanol in pump A, and phosphate buffer (pH 3) in pump B. The system was operated at 1.5 ml/min flow rate with fixed ratios of solvent A and solvent B (70:30) for 15 min. Twenty microliters of the sample were injected through a SIL-20A HTC Prominence autosampler, and the column oven temperature was maintained at 40 °C. The exact quantities of the identified ingredients were quantified by comparing the area under the curve (AUC) of corresponding reference standards.

In the outcomes of HPLC analysis, rutin and gallic acid were identified as chief active ingredients with a concentration of 0.375 mg and 0.0328 mg, respectively in 5 mg of MeMC. The HPLC chromatogram and identification and quantification of active compounds from MeMC are given in Supplementary data 1.

***Experimental animals***

Six weeks old male C57BL/6 mice were procured from Vivo Biosciences, Hyderabad, India. Animals were housed in polypropylene cages at a temperature of 23 ^°^C ± 1^°^C and relative humidity of 45% to 55% in a clean environment under a 12-hr light/dark cycle. All animals were fed diet and water *ad libitum* during the experimental period. After adaptation for a week, mice were fed either chow diet (10% Kcal, Research Diet Inc, New Jersey, D10001) or high-fat diet (HFD) (60% kcal from fat; Research Diet Inc., New Jersey, USA, D12492) for 10 weeks. 

All experimental protocols involving animals were approved by Institutional Animal Ethics Committee (IAEC) of Ms. Connexios Life Sciences Pvt. Ltd., Bangalore (CPCSEA N0 1241/bc/CPCSCEA), and all experiments were conducted as per principles and guidelines of the Committee for the Purpose of Control and Supervision of Experimentation on Animals (CPCSEA), India.

***Acute oral toxicity studies***

Acute oral toxicity for MeMC was determined as per OECD guideline no. 425, and LD50 was calculated using AOT425 stat program (OECD guidelines no.425, 2001) (27).

***Study design***

In the present study, diet-induced obesity (DIO) mouse model was developed by feeding the HFD to C57BL/6 mice. The development of hyperglycemic state was confirmed by checking the fasting blood glucose at one-week regular intervals. 

**Figure 1:**
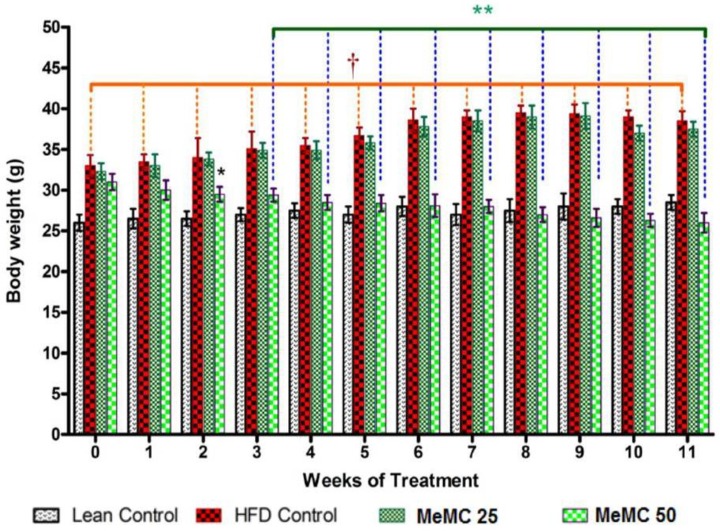
Effect of MeMC on high fat diet-induced altered body weight in C57 mice

HFD- High fat diet, MeMC- Methanolic fruit extract of *Mommordica cymbalaria *

All the values are expressed as mean± SEM (n=10); means of various groups were statistically compared by one way ANOVA followed by Dunnett’s multiple comparison test using Graph Pad version 5.0. The HFD control group animals showed significant increase in body weight compared to Lean control (*P*<0.01). The MeMC (25 & 50 mg/kg) treatment showed significant reduction in body weight compared to HFD group (*P*<0.01)

**Figure 2:**
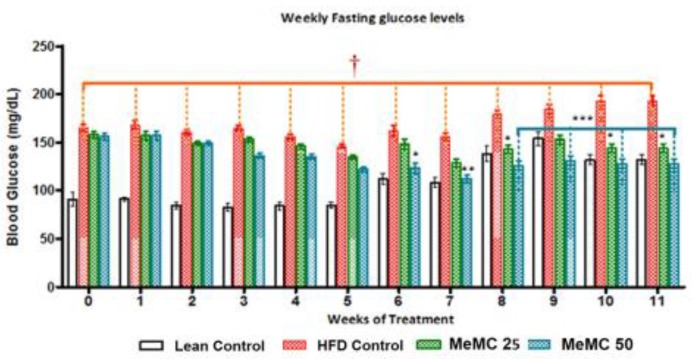
Effect of MeMC on fasting blood glucose levels in high fat diet fed C57 mice

HFD- High fat diet, MeMC- Methanolic fruit extract of *Mommordica cymbalaria *

All the values are expressed as mean±SEM (n=10); means of various groups were statistically compared by one way ANOVA followed by Dunnett’s multiple comparison test using Graph Pad version 5.0. The HFD control group animals showed significant increase in blood glucose levels compared to Lean control (*P*<0.01). The MeMC (25 & 50 mg/kg) treatment from week-6 onwards showed significant reduction in blood glucose levels compared to HFD group (*P*<0.01), indicating the anti-diabetic property of MeMC

**Figure 3:**
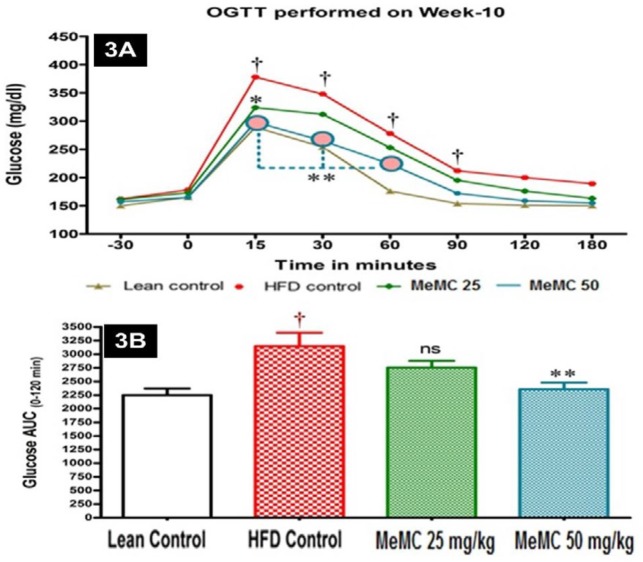
Effect of MeMC on oral glucose tolerance in High fat diet fed C57 mice

HFD- High fat diet, MeMC- Methanolic fruit extract of *Mommordica cymbalaria *

All the values are expressed as mean± SEM (n = 8); means of various groups were statistically compared by one way ANOVA followed by Dunnett’s multiple comparison test using Graph Pad version 5.0. Figure 3A. Oral glucose tolerance test - The HFD control group animals showed significant increase in the accumulation of glucose (glucose tolerance) compared to Lean control (*P*<0.01). However, the MeMC (50 mg/kg) treatment has cleared the blood glucose significantly compared to HFD group (*P*<0.01). Figure 3B: AUC of Oral Glucose tolerance test – The AUC of HFD group was significantly higher compared to lean control indicating the high glucose intolerance of HFD group. However, the MeMC (50 mg/kg) treatment has shown lower AUC compared to HFD group (*P*<0.01), indicating the ameliorative potential of MeMC against HFD-induced glucose intolerance

**Figure 4:**
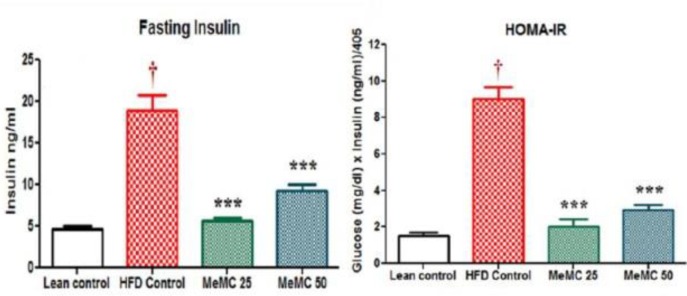
Effect of MeMC on fasting insulin levels and HOMA-IR index

HFD- High fat diet, MeMC- Methanolic fruit extract of* Mommordica cymbalaria *

All the values are expressed as mean±SEM (n=10); means of various groups were statistically compared by one way ANOVA followed by Dunnett’s multiple comparison test using Graph Pad version 5.0. The HFD control group animals showed significant increase in serum insulin levels (*P*<0.01), and significant increase in HOMA-IR compared to lean control group, as a sign of insulin resistance. The MeMC (25 and 50 mg/kg) showed significantly less concentration of serum insulin along with less HOMA-IR index compared to HFD control (*P*<0.01)

**Figure 5:**
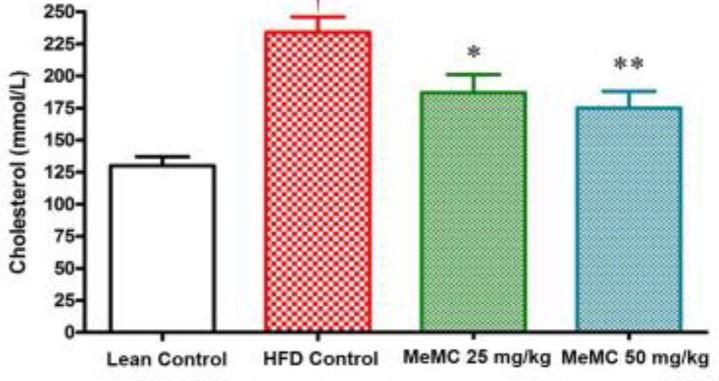
Effect of MeMC on serum cholesterol levels in high fat diet fed C57 mice

HFD- High fat diet, MeMC- Methanolic fruit extract of *Mommordica cymbalaria*

All the values are expressed as mean±SEM (n=10); means of various groups were statistically compared by one way ANOVA followed by Dunnett’s multiple comparison test using Graph Pad version 5.0. The HFD control group animals showed significant increase in serum cholesterol levels compared to Lean control (*P*<0.01). The MeMC (25 & 50 mg/kg) treatment showed significant reduction in serum cholesterol levels compared to HFD group (*P*<0.05 & *P*<0.01)

**Figure 6:**
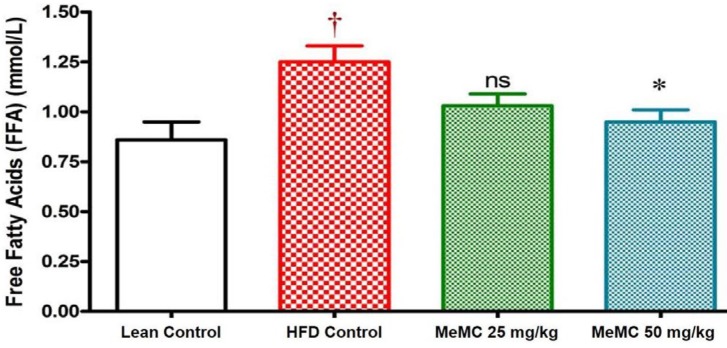
Effect of MeMC on serum free fatty acid levels in high fat diet fed C57 mice

HFD- High fat diet, MeMC- Methanolic fruit extract of *Mommordica cymbalaria *

All the values are expressed as mean±SEM (n=10); means of various groups were statistically compared by one way ANOVA followed by Dunnett’s multiple comparison test using Graph Pad version 5.0. The HFD control group animals showed significant increase in serum free fatty acid levels compared to Lean control (*P*<0.01). The MeMC (50 mg/kg) treatment showed significant reduction in serum free fatty acid levels compared to HFD group (*P*<0.05)

**Figure 7:**
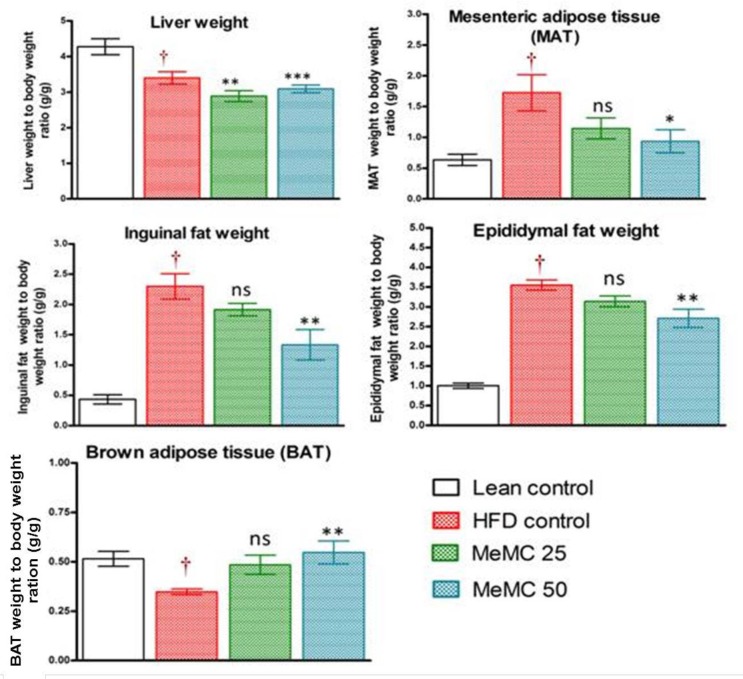
Effect of MeMC on organ weights in High fat diet fed C57 mice

HFD- High fat diet, MeMC- Methanolic fruit extract of *Mommordica cymbalaria *

All the values are expressed as mean±SEM (n=10); means of various groups were statistically compared by one way ANOVA followed by Dunnett’s multiple comparison test using Graph Pad version 5.0. The HFD control group animals showed significant increase in liver weight (*P*<0.01), MAT (*P*<0.01), Inguinal fat (*P*<0.01), Epididymal fat (*P*<0.01) weight and decreased the BAT (*P*<0.01) weight compared to lean control. The MeMC (50 mg/kg) treatment has significantly increased the BAT weight (*P*<0.01) and significantly reduced the other all tissue weights compared to HFD control (*P*<0.01)

**Figure 8:**
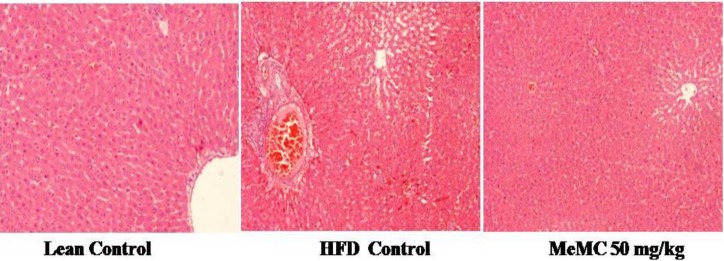
Effect of MeMC on the High fat diet-induced histological changes of the liver in C57 mice

Illustration showing histopathology of liver of various groups. Lean control–Showing normal cytoarchitecture, HFD control–Showing the abnormal cellular changes such as fatty infiltration, macrophage infiltration, hypertrophy and vascular changes of hepatocytes due to high fat diet, MeMC 50 mg/kg- Showing very minimal histopathological lesions (H&E x100)

*Grouping of animals and treatment*

Following high-fat diet for 11 weeks, the animals were randomized in to specific treatment groups, based on the body weight, glucose AUC during OGTT, and fasting blood glucose levels. The study animals were assigned into four groups (G1 to G4, n=10), G1 and G2 served as lean control and HFD control, while G3 and G4 received MeMC 25 mg/kg and 50 mg/kg doses, respectively. The 25 and 50 mg/kg doses of MeMC were selected based on the literature reports and pilot study conducted in-house. 

The animals in the lean control group (n = 10) were fed normal chow diet, while animals in the HFD control and MeMC treatment groups were fed high-fat diet throughout the experimental period, and all treatments were given once a day orally for a period of 11 weeks.

*Formulation and administration of the extract*

The methanolic extract of *M. cymbalaria *was suspended in 3% Tween 80 and administered orally at a dose volume of 5 ml/kg, the animals in lean control and HFD control received 3% Tween 80 in distilled water at a dose volume of 5 ml/kg throughout the study.

***Evaluation parameters***

*Body weight*

The body weight was recorded once weekly, from the day of study initiation to the termination.

*Blood glucose measurement*

All animals were fasted for 6 hr, and fasting blood glucose levels were measured using Accu-check glucometer (Roche Diagnostics), every week until the end of the study.

*Oral glucose tolerance test (OGTT)*

This test was performed on the week 10 of the study; in brief, 2 g/kg body weight of glucose load was administered by the oral route and OGTT was determined by measuring the blood glucose levels at different time intervals viz., -30, 0, 30, 60, 120, and 180 min using an Accu-check glucometer; the blood samples were collected from the tail vein. The values were expressed as mean ± SEM, and a graph of time in minutes vs glucose concentration was plotted and area under the curve (AUC) was determined.

*Estimation of total cholesterol, glycerol and free fatty acid (FFA)*

After 11 weeks of treatment, all animals were fasted for 6 hr, and under isoflurane anesthesia, blood samples were collected by retro-orbital puncture and allowed to clot for 30 min at room temperature, centrifuged at 10000 rpm for 10 min at 4 ^°^C, and the serum was collected for analysis. 

The serum total cholesterol was measured using ERBA diagnostic kit (ERBA Mannheim, Mannheim, Germany) with the help of EM360 auto-analyzer (Transasia Bio-Medicals Ltd); Glycerol and FFA were estimated by Colorimetric method using commercially available Free Fatty acid analysis kit (Sigma-Aldrich, Bangalore, India) and Randox kit (Randox Laboratories Ltd, United Kingdom), respectively.

*Estimation of serum insulin*

Serum levels of insulin were determined by using Mercodia mouse insulin ELISA kit (Mercodia AB, Uppsala, Sweden), as per the user manual provided by the manufacturer.

*Assessment of insulin resistance*

Using serum insulin and corresponding blood glucose values, insulin resistance can be calculated by a well-known and most widely used model known as Homeostatic model assessment of Insulin Resistance (HOMA-IR).

*Necropsy and organ weights*

At the end of the study, all animals were fasted overnight, body weight was recorded and under deep ether anesthesia all animals were euthanized and organs such as liver, mesenteric adipose tissue (MAT), brown adipose tissue (BAT), epididymal fat and inguinal fat were collected and weighed. The organ weights were expressed with respect to body weight (organ weight to body weight ratio).

*Histopathology*

After measuring the weight, the liver tissue was fixed in 10% neutral buffered formalin and subjected for histopathological evaluation. In short, 4 µm thickness slices of liver tissue were obtained using a microtome instrument (Leica RM2235 rotary microtome, Leica Biosystems, Mumbai, India) and the sections were stained with hematoxylin and eosin (H&E) and subjected to evaluation.

***Statistical analysis***

All experimental findings were expressed as mean ± SEM, and they were statistically analyzed using one-way ANOVA followed by Dunnett’s multiple comparisons test. HOMA-IR index was calculated by using the standard formula obtained from the literature, and histopathological scores were statistically compared using the Kruskal-Wallis test (non-parametric test) followed by Dunn’s Multiple Comparisons Test. All statistical analysis was performed using GraphPad Prism ver. 5.0 for Windows (GraphPad Software, San Diego, California, USA). The minimum level of significance was fixed at *P*< 0.05.

## Results

***Extraction of plant material***

The extractive yield of methanolic extract (MeMC) was found to be 3.2 % W/W

***Acute oral toxicity study***

The outcome of this study revealed that MeMC is safe up to 2000 mg/kg, PO. Further, no signs of toxicity were observed either in the short term (48 hr) or in the long-term (14 days) observation period.

***Development of diabetes and insulin resistance in C57BL/6 mice***

Feeding HFD to male C57BL/6 mice for 10 weeks resulted in significant rise in body weight (2 fold increase), blood glucose (hyperglycemia), and insulin (hyperinsulinemia) compared to the lean control group, and also the HFD feeding resulted in impaired oral glucose tolerance. This condition exactly mimics the diabetes and insulin resistance developed in humans. These animals were randomized into 4 groups based on body weight, AUC of OGTT, and fasting blood glucose.

***Effect of MeMC on body weight***

MeMC (50 mg/kg) has shown a significant decrease in the body weight from week-2 onwards compared to HFD control (*P*<0.01). In contrary, the low dose (25 mg/kg) of MeMC did not show a significant effect on the body weight compared to HFD control (Figure 1).

***Effect of MeMC on the glycemic index***

The MeMC (50 mg/kg) treated group showed a significant decrease in blood glucose levels from week 6, after treatment initiation, onwards. Interestingly, from week 8 onwards both doses of MeMC (25 & 50 mg/kg) showed a further decrease in the blood glucose levels compared to HFD control (*P*<0.05, *P*<0.001) (Figure 2).

***Effect of MeMC on OGTT***

In this test, the area under the curve (AUC) obtained is inversely proportional to extent of glucose tolerance. In the present study, the HFD control animals showed higher AUC as an indication of glucose intolerance. However, MeMC (50 mg/kg) treatment showed a significant improvement in glucose tolerance, and it was statistically significant compared to HFD control (*P*<0.01), the results are depicted in Figures 3A and 3B.

***Effect of MeMC on fasting insulin levels and insulin resistance***

In the present study, the HFD control animals showed a significant increase in serum insulin levels compared to lean control (*P*<0.01), as a cardinal sign of hyperinsulinemia. However, the MeMC (50 mg/kg) treated group showed significantly low levels of insulin compared to HFD control (*P*<0.01). Further, hyperinsulinemia and HOMA-IR are two parameters which have a positive correlation with each other and represent insulin resistance, in the present study the HFD control animals showed very high levels of HOMA-IR index compared to lean control (*P*<0.01), which is considered as a hallmark of insulin resistance state. On the other hand, MeMC (50 mg/kg) treatment showed lower HOMA-IR index compared to HFD control (*P*<0.01), as an indication of good insulin sensitivity or low insulin resistance. The results are given in Figures 4A and 4B.

***Effect of MeMC on cholesterol and FFA levels***

In the present study, the HFD control animals showed a significant increase in serum cholesterol and FFA levels compared to lean control (*P*<0.01). Interestingly, MeMC (50 mg/kg) reduced the serum levels of cholesterol and FFA significantly compared to HFD control (*P*<0.01). The results are given in Figures 5 and 6.

***Effect of MeMC on liver and adipose tissue weight***

In the present study, various tissue weights were taken and expressed with respect to body weight (organ weight to body weight ratio). Particularly, in the HFD control group, a significant increase in liver weight and different adipose tissue weights were observed compared to lean control (*P*<0.01). However, the MeMC (50 mg/kg) treatment ameliorated all these changes and maintained the weight of the liver, MAT, epididymal, and inguinal fat, significantly less than the HFD control (*P*<0.01) and increased the BAT weight compared to HFD control (*P*<0.01). The results are given in Figure 7.

***Effect of MeMC on HFD induced hepatocellular damage***

 In line with all other findings, histopathology of liver tissue showed significant fatty infiltration, macrophage infiltration, vascular changes, and hypertrophy of hepatocytes in the HFD control group; in contrast, the lean control animals showed normal cytoarchitecture of the liver. Interestingly, MeMC 50 mg/kg treated animals showed very mild fatty infiltration without major changes in the histoarchitecture of hepatocytes (Figure 8). The histopathological scoring and statistical analysis of histopathology data are given in Supplementary data 2. 

## Discussion

In the present study, MeMC was evaluated against HFD-induced diabetes and insulin resistance in C57BL/6 mice, the outcomes have demonstrated that MeMC possesses potent antidiabetic activity and also reversed the insulin resistance state developed due to a high-fat diet.

The C57BL/6 mouse model used in the present study is considered to very closely mimic the progression of obesity and diabetes in humans (28). Therefore, this model is the most widely used and globally accepted model, relevant to diet-induced obesity (29). In literature, it is reported that the C57BL/6 mice when fed with HFD* ad libitum*, develop obesity, hyperglycemia, and hyperinsulinemia along with hypertension, however, they remain lean without metabolic abnormalities when fed normal chow diet *ad libitum* (30), the metabolic changes observed in HFD control are associated with significant increase in liver weight (metabolic organ), increase in white adipose tissue weight (epididymal, inguinal, mesenteric, and others), and decrease in the brown adipose tissue weights (28, 30)**.**

In agreement with the literature reports, in the present study, the HFD control animals showed a significant increase in body weight, blood glucose, and insulin levels compared to lean control. The HFD control animals showed a significant increase in the AUC of oral glucose tolerance test and high levels of insulin. In short, although there is enough insulin in the systemic circulation, there was not enough glucose clearance from the blood, which is otherwise called insulin resistance state (31). Further, the HOMA-IR index is one of the most widely used methods for expressing insulin resistance (32). In present study, the HFD control animals showed hyperinsulinemia along with significant increase in HOMA-IR index as an indication of insulin resistance, along with these changes the HFD control animals also showed significant elevation of total cholesterol and free fatty acid levels as a signs of hyperlipidemia, which further lead to accumulation of glycerol and fatty acids in the liver and result in fatty liver, and hence increase the liver weight; in support of this, histopathology of liver showed significant fatty infiltration, macrophage infiltration, and vascular changes along with hepatocellular hypertrophy. In addition, evidently clear increase in liver weight, and increase in white adipose tissue weight (inguinal, epididymal, and mesenteric) was observed along with a decrease in the brown adipose mass (BAT). 

Interestingly, MeMC (50 mg/kg) treatment alleviated all these metabolic changes (body weight, hyperglycemia, hyperinsulinemia, hyperlipidemia, and altered organ weights) that occurred due to HFD. 

Altogether, with these observations, we can conclude that MeMC elicits these actions by sensitizing the insulin action, therefore with minimum insulin concentration the glucose clearance was high. Furthermore, MeMC also showed a decrease in cholesterol and FFA levels, with a decrease in liver and white adipose weight, and a significant increase in the BAT weight compared to HFD, also the histopathology of MeMC treated group showed very minimal fatty infiltration in the hepatocytes without other changes. In fact, increase in BAT is considered a good sign of better metabolic rate, hence with all these observations put together, it can be hypothesized that the metabolic rate and insulin responsiveness was better in the MeMC treated group compared to HFD control. 

In line with the outcomes of the present study, in our previous study, we found that MeMC could enhance glucose uptake and also showed increased expression of adiponectin, leptin, PPAR-γ, and GLUT-4 gene expression *in vitro *(25-26)**.** In literature, the role of these markers in the development of diabetes and insulin has been well documented. In short, PPAR-γ is a nuclear factor which binds with the PPAR-responsive element (PPRE) and increases the adiponectin promoter activity in adipocytes (33). Adiponectin is the most enormously expressed adipokine and is well known for its insulin-sensitizing action; through adiponectin receptors (AdipoR1 and AdipoR2) it activates AMPK, PPAR-α, and other unknown pathways, to enhance insulin sensitivity. Thus adiponectin has a positive correlation with insulin sensitivity (34). In skeletal muscles, adiponectin increases the expression of CD36 and acyl-coenzyme-A oxidase, and thereby reduces the free fatty acid and triglyceride levels, which in turn contribute to improved insulin signaling transduction (35). Further, adiponectin also stimulates phosphorylation of acetyl coenzyme-A carboxylase (ACC) and activates AMPK, which results in enhanced glucose uptake, increased fatty acid combustion, reduced gluconeogenesis, and all these changes together contribute to reduced blood glucose levels (36). Besides, leptin is yet another chief adipocytokine known to play a pivotal role in maintaining the body weight, metabolism and reproductive functions (37), leptin largely exists in the central nervous system and is known for its predominant role in regulating food consumption, specifically lesser levels of leptin increase food consumption; it is well manifested in leptin knockout animals (38), and also it is well known that obese individuals have very low leptin levels as compared to healthy volunteers (39).

In our previous studies we observed that MeMC could enhance the expression of adiponectin, leptin, PPAR-γ, and GLUT-4 genes and increase glucose uptake (25, 26), thus, the mechanism behind the antidiabetic and insulin sensitizer activity of MeMC observed in the present study is thought to be mediating through enhanced expression of adiponectin, leptin, PPAR-γ, and GLUT-4 genes. Further, the HPLC analysis of MeMC revealed the presence of gallic acid and rutin as chief ingredients; relevant to the present study gallic acid (40, 41), and rutin (42-44) are scientifically well proven to possess potential antidiabetic activity.

## Conclusion

With the outcomes of the present study, we can conclude that MeMC possesses potential antidiabetic and insulin-sensitizing activity by ameliorating the HFD induced body weight gain, hyperglycemia, hyperinsulinemia, hyperlipidemia, and increased HOMA-IR index along with maintaining the various organ weights near the normal range. 

## Conflicts of Interest

Authors declare that there are no conflicts of interest.

## References

[1] American Diabetes Association (2010). Diagnosis and classification of diabetes mellitus. Diabetes Care.

[2] Wild S, Roglic G, Green A, Sicree R, King H (2004). Global prevalence of diabetes estimates for 2000 and projections for 2030. Diabetes care.

[3] Liu JP, Zhang M, Wang WY, Grimsgaard S (2004). Chinese herbal medicines for type 2 diabetes mellitus. Cochrane Database Syst Rev.

[4] Modak M, Dixit P, Londhe J, Ghaskadbi S, Devasagayam TP (2007). Indian herbs and herbal drugs used for the treatment of diabetes. J Clin Biochem Nutr.

[5] Dwivedi S, Daspaul A (2013). Antidiabetic herbal drugs and polyherbal formulation used for diabetes: A review. J Phytopharmacol.

[6] Chawla R, Thakur P, Chowdhry A, Jaiswal S, Sharma A, Goel R, Sharma J, Priyadarshi SS, Kumar V, Sharma RK, Arora R (2013). Evidence based herbal drug standardization approach in coping with challenges of holistic management of diabetes: a dreadful lifestyle disorder of 21st century. J Diabetes Metab. Disord.

[7] Wadood A, Wadood N, Shah SA (19889). Effects of Acacia arabica and Caralluma edulis on blood glucose levels on normal and alloxan diabetic rabbits. J Pak Med Assoc.

[8] Karunanayake EH, Welihinda J, Sirimanne SR, Sinnadorai G (1984). Oral hypoglycemic activity of some medicinal plants of Sri Lanka. J Ethnopharmacol.

[9] Kumari K, Mathew BC, Augusti KT (1995). Antidiabetic and hypolipidaemic effects of S-methyl cysteine sulfoxide, isolated from Allium cepa Linn. Indian J Biochem Biophys.

[10] Chattopadhyay RR, Chattopadhyay RN, Nandy AK, Poddar G, Mitra SK (1987). Preliminary report on anti- hyperglycemic effect of fraction of fresh leaves of Azadiracta indica (Beng neem). Bull Calcutta Sch Trop Med.

[11] Chattopadhyay RR, Chattopadhyay RN, Nandy AK, Poddar G, Mitra SK (1987). The effect of fresh leaves of Azadiracta indica on glucose uptake and glycogen content in the isolated rat hemidiaphragm. Bull Calcutta Sch Trop Med.

[12] Pari L, Amarnath SM (2004). Antidiabetic activity of Boerhavia diffusa L effect on hepatic key enzymes in experimental diabetes. J Ethnopharmacology.

[13] Dhanabal SP, Sureshkumar M, Ramanathan M, Suresh B (2005). Hypoglycemic effect of ethanolic extract of Musa sapientum on alloxan induced diabetes mellitus in rats and its relation with antioxidant potential. J Herb Pharmacother.

[14] Pari L, Umamaheswari J (2000). Antihyperglycaemic activity of Musa sapientum flowers: effect on lipid peroxidation in alloxan diabetic rats. Phytother Res.

[15] Sabu MC, Kuttan R (2002). Antidiabetic activity of medicinal plants and its relationship with their antioxidant property. J Ethnopharmacol.

[16] Adallu B, Radhika B (2000). Hypoglycemic, diuretic and hypocholesterolemic effect of winter cherry (Withania somnifera, Dunal) root. Ind J Exp Biol.

[17] Rao BK, Kesavulu MM, Giri R, Appa Rao C (1999). Antidiabetic and hypolipidemic effects of Momordica cymbalaria Hook fruit powder in alloxan-diabetic rats. J Ethnopharmacol.

[18] Rao BK, Kesavulu MM, Giri R, Appa Rao C (2001). Antihyperglycemic activity of Momordica cymbalaria in alloxan diabetic rats. J Ethnopharmacol.

[19] Rao BK, Kesavulu MM, Appa Rao C (2003). Evaluation of antidiabetic effect of Momordica cymbalaria fruit in alloxan-diabetic rats. Fitoterapia.

[20] Prashanth SJ, Suresh D, Maiya PS (2013). In vitro antioxidant studies of Momordica cymbalaria. Asian J BioScie.

[21] Dhasan PB, Jegadeesan M, Kavimani S (2010). Antiulcer activity of aqueous extract of fruits of Momordica cymbalaria Hook f in Wistar rats. Pharmacog Res.

[22] Marella S, Maddirela DR, Badri KR, Jyothi Kumar MV, Chippada A (2015). Antihyperlipidemic and biochemical activities of Mcy protein in streptozotocin induced diabetic rats. Cell Physiol Biochem.

[23] Murthy NS, Snehalatha N, Anuradha N, Seetharam YN (2007). Anticonvulsant activity of ethanolic extract of Momordica cymbalaria Fenzlex N aud. Focus Altern Complement Ther.

[24] Koneri RB, Samaddar S, Simi SM, Rao ST (2014). Neuroprotective effect of a triterpenoid saponin isolated from Momordica cymbalaria Fenzl in diabetic peripheral neuropathy. Ind J Pharmacol.

[25] Mahesh Kumar P, Venkataranganna MV, Manjunath K, Viswanatha GL, Ashok G (2014). Methanolic extract of Momordica cymbalaria enhances glucose uptake in L6 myotubes in vitro by up-regulating PPAR-γ and GLUT-4. Chin J Nat Med.

[26] Mahesh Kumar P, Venkataranganna MV, Manjunath K, Viswanatha GL, Ashok G (2016). Methanolic fruit extract of Momordica cymbalaria alleviates insulin resistance by enhancing expression of adiponectin and leptin in vitro. World J Pharm Pharm Sci.

[27] OECD 2001 Guideline on acute oral toxicity (AOT) Environmental health and safety monograph series on testing and adjustment No.425. http://www.oecd.org/chemicalsaf%20e%20t%20y/r%20i%20s%20k%20-assessment/1948378.pdf.

[28] Speakman J, Hambly C, Mitchell S, Krol E (2007). Animal models of obesity. Obes Rev.

[29] Johnson PR, Greenwood MR, Horwitz BA, Stern JS (1991). Animal models of obesity: genetic aspects. Annu Rev Nutr.

[30] Wang CY, Liao JK (2012). A mouse model of diet-induced obesity and insulin resistance. Methods Mol Biol.

[31] Gisela W (2005). Insulin and insulin resistance. Clin Biochem Rev.

[32] Wallace TM, Levy JC, Matthews DR (2004). Use and Abuse of HOMA Modeling. Diabetes Care.

[33] Iwaki M, Matsuda M, Maeda N, Funahashi T, Matsuzawa Y, Makishima M, Shimomura I (2003). Induction of adiponectin, a fat-derived antidiabetic and antiatherogenic factor, by nuclear receptors. Diabetes.

[34] Bouskila M, Pajvani UB, Scherer PE (2005). Adiponectin: a relevant player in PPAR-γ agonist mediated improvements in hepatic insulin sensitivity?. Int J Obes.

[35] Ronti T, Lupattelli G, Mannarino E (2006). The endocrine function of adipose tissue: an update. Clin. Endocrinol.

[36] Zheng F, Zhang S, Lu W, Wu F, Yin X, Yu D, Pan Q, Li H (2014). Regulation of insulin resistance and adiponectin signaling in adipose tissue by liver X receptor activation highlights a cross-talk with PPARγ. PLoS One.

[37] Flier JS (2004). Obesity wars: molecular progress confronts an expanding epidemic. Cell.

[38] Zhang Y, Proenca R, Maffei M, Barone M, Leopold L, Friedman JM (1994). Positional cloning of the mouse obese gene and its human homologue. Nature.

[39] Farooqi IS, Matarese G, Lord GM (2002). Beneficial effects of leptin on obesity, T cell hyporesponsiveness, and neuroendocrine/metabolic dysfunction of human congenital leptin deficiency. J Clin Invest.

[40] Snehal SP, Goyal RK (2011). Cardioprotective effects of gallic acid in diabetes-induced myocardial dysfunction in rats. Pharmacog Res.

[41] Li L, Chen H, McGee SL (2008). Mechanism of AMPK regulating GLUT4 gene expression in skeletal muscle cells. Sheng Wu Yi Xue Gong Cheng Xue Za Zhi.

[42] Cai Y, Fan C, Yan J (2012). Tian NX Effects of rutin on the expression of PPARγ in skeletal muscles of db/db mice. Planta Med.

[43] Hsu CY, Shih HY, Chia YC, Lee CH, Ashida H, Lai YK, Weng CF (2014). Rutin potentiates insulin receptor kinase to enhance insulin-dependent glucose transporter 4 translocation. Mol Nutr Food Res.

[44] Naowaboot J, Chung CH, Choi R (2015). Rutin stimulates adipocyte differentiation and adiponectin secretion in 3T3-L1 adipocytes. J Med Assoc Thailand.

